# *Trypanosoma brucei gambiense* Group 1 Is Distinguished by a Unique Amino Acid Substitution in the HpHb Receptor Implicated in Human Serum Resistance

**DOI:** 10.1371/journal.pntd.0001728

**Published:** 2012-07-10

**Authors:** Rebecca E. Symula, Jon S. Beadell, Mark Sistrom, Kehinde Agbebakun, Oliver Balmer, Wendy Gibson, Serap Aksoy, Adalgisa Caccone

**Affiliations:** 1 Department of Ecology and Evolutionary Biology, Yale University, New Haven, Connecticut, United States of America; 2 Yale School of Public Health, Division of Epidemiology and Public Health, New Haven, Connecticut, United States of America; 3 Department of Biology, University of Mississippi, Mississippi, United States of America; 4 Swiss Tropical and Public Health Institute, Basel, Switzerland; 5 Institute of Zoology, University of Basel, Basel, Switzerland; 6 Research Institute of Organic Agriculture, Frick, Switzerland; 7 School of Biological Sciences, University of Bristol, Bristol, United Kingdom; International Centre of Insect Physiology and Ecology, Kenya

## Abstract

*Trypanosoma brucei rhodesiense* (*Tbr*) and *T. b. gambiense* (*Tbg*), causative agents of Human African Trypanosomiasis (sleeping sickness) in Africa, have evolved alternative mechanisms of resisting the activity of trypanosome lytic factors (TLFs), components of innate immunity in human serum that protect against infection by other African trypanosomes. In *Tbr*, lytic activity is suppressed by the *Tbr-*specific serum-resistance associated (SRA) protein. The mechanism in *Tbg* is less well understood but has been hypothesized to involve altered activity and expression of haptoglobin haemoglobin receptor (HpHbR). HpHbR has been shown to facilitate internalization of TLF-1 in *T.b. brucei* (*Tbb*), a member of the *T. brucei* species complex that is susceptible to human serum. By evaluating the genetic variability of *HpHbR* in a comprehensive geographical and taxonomic context, we show that a single substitution that replaces leucine with serine at position 210 is conserved in the most widespread form of *Tbg* (*Tbg* group 1) and not found in related taxa, which are either human serum susceptible (*Tbb*) or known to resist lysis via an alternative mechanism (*Tbr* and *Tbg* group 2). We hypothesize that this single substitution contributes to reduced uptake of TLF and thus may play a key role in conferring serum resistance to *Tbg* group 1. In contrast, similarity in *HpHbR* sequence among isolates of *Tbg* group 2 and *Tbb*/*Tbr* provides further evidence that human serum resistance in *Tbg* group 2 is likely independent of HpHbR function.

## Author Summary

Human African Trypanosomiasis, or sleeping sickness, is caused by two different parasites: *Trypanosoma brucei gambiense* (*Tbg*) and *T. b. rhodesiense* (*Tbr*). Each parasite employs a different mechanism to resist trypanosome lytic factor (TLF), the active innate immune component of human serum. In *Tbg* group 1, which causes the vast majority of disease cases, the mechanism is thought to involve the reduced activity of a receptor involved in binding and internalizing TLF. In this study, we investigate genetic variation in this receptor across a broad geographic sample of *Tbg* and closely related trypanosomes to test whether unique polymorphisms in the receptor from *Tbg* may explain its altered function. We identified a single mutation in all copies of the receptor gene sequenced from *Tbg* but not in any other closely related species. This finding suggests that this single mutation could play a key role in conferring human infectivity to *Tbg*. Given the possible consequences for drug development and diagnostics, we suggest that future functional studies target this mutation to fully elucidate its role.

## Introduction

**Figure 1:**
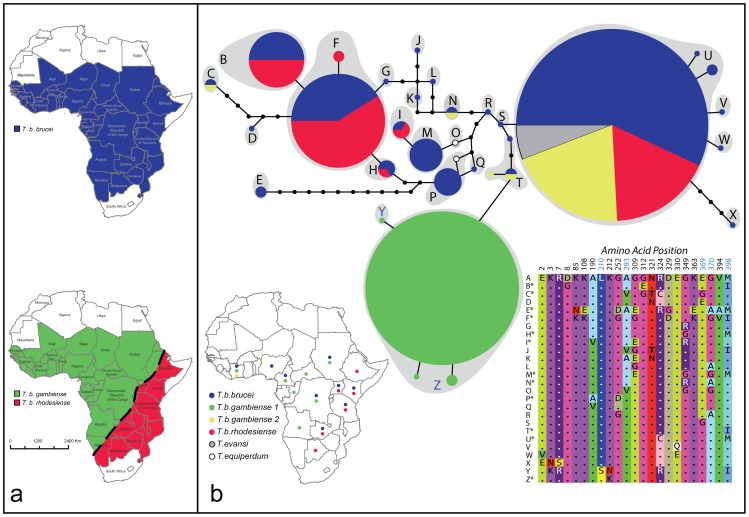
*Trypanosoma brucei* distribution, sampling scheme and relationships among HpHbR DNA and amino acid sequences. a. Approximate geographic distribution of the animal-restricted parasite *T. b. brucei* (blue) and the human-infective parasites *T. b. gambiense* groups 1 and 2 (*Tbg*1, *green*; *Tbg*2, yellow) and *T. b. rhodesiense* (*Tbr*; red) which cause human African trypanosomiasis. b. A haplotype network (top) shows the relationships among unique HpHbR alleles (represented by colored circles (sampled) or black dots (unsampled)) and highlights the differentiation of *Tbg*1 from other taxa at this locus. Each line in the network represents one nucleotide change. Circle size is proportional to allele frequency. Colored sections of the circles indicate the relative frequency with which a particular allele was recovered from different taxa within the subgenus *Trypanozoon* (key at bottom left). Dots on the map indicate the country where isolates of each taxon, as shown by color, were collected. Grey shading in the network joins alleles with an identical inferred amino acid sequence. Unique amino acid sequences found in this study are identified by a capital letter in the haplotype network and a corresponding letter in the alignment of variable positions (bottom right). Reference sequence A, previously identified in *Tbb* strain Lister 427 (Kieft et al. 2010) was not recovered in this study. Asterisks indicate amino acid sequences found in more than one isolate. Amino acids in the alignment are represented with standard single letter codes and color-coded for ease of comparison across sequences. The two amino acid sequences recovered from *Tbg*1 (Y and Z) share a single substitution at position 210 that was not found in any other taxa. Amino acid positions labeled in blue correspond to positions previously identified as playing a possible role in altered activity of HpHbR in *Tbg*1 (Kieft et al. 2010).

Trypanosomiasis, a deadly disease of humans and livestock in sub-Saharan Africa, is caused by protozoan parasites of the genus *Trypanosoma*, which are transmitted between mammalian hosts by insect vectors of the genus *Glossina* (tsetse). Human-infective members of the *Trypanosoma brucei* complex cause the human form of the disease, Human African Trypanosomiasis (HAT), or sleeping sickness. *T. b. rhodesiense* (*Tbr*) causes an acute form of HAT in eastern Africa, while *T. b. gambiense* group 1 (*Tbg*1) causes a chronic form of the disease in western and central Africa and accounts for over 90% of reported cases (Figure 1a). *T. b. gambiense* group 2 (*Tbg*2), a rare form described from West Africa in the 1970s and 1980s, also causes human disease but the trait of human-infectivity is not stable [1], [2], [3]. The final member of the *brucei* complex, *T. b. brucei* (*Tbb*), is not infective to humans, but, together with other animal trypanosome species, causes the livestock wasting disease, Nagana, across a range that overlaps with that of the human-infective parasites.

Humans possess an innate resistance to some trypanosomes through the action of trypanosome lytic factors (TLFs) in their serum [4]. TLF-1 is a high-density lipoprotein complex that includes the active toxin apolipoprotein L-I (apoL-I) in association with haptoglobin-related protein (Hpr). In the primary immune pathway [5], [6], [7], TLF-1 is bound and internalized via a haptoglobin haemoglobin receptor (HpHbR) on the surface of susceptible trypanosomes. Uptake of TLF-1 is followed by disruption of the lysosomal membrane by apoL-I and eventual cell lysis. While *Tbb* is susceptible to lysis by human TLF-1, *Tbr*, *Tbg*1 and *Tbg*2 are resistant. In *Tbr*, the serum-resistance associated (SRA) protein confers resistance to TLF-1 [8] by binding directly to apoL-I after it has been internalized into the cell, inhibiting its lysosome-lytic capacity [9]. *Tbg*1 and *Tbg*2, on the other hand, lack the gene encoding SRA and are thought to have evolved an independent mechanism to prevent lysis by TLF.

In *Tbg*2, apoL-I is also internalized, but lysis is prevented by an unidentified mechanism [10]. In *Tbg*1, the mechanism is better understood and appears to involve reduced expression and altered function of the parasite HpHbR [11]. Sequencing of a few isolates of *Tbg* and *Tbb* led Kieft et al. [11] to suggest that mutations in *HpHbR* may have altered TLF-1 binding in *Tbg1*. Specifically, the authors identified five non-synonymous substitutions shared by the four sequenced isolates of *Tbg1*, but not present in two *Tbb* isolates. This work has helped to narrow the universe of possible structural differences in HpHbR that could, for example, eventually be exploited to design novel drugs to overcome *Tbg1* resistance. However, the small number of isolates examined to date is not sufficient to determine whether the mutations are really *Tbg1*-specific. While genetic variation in *Tbg*1 is extremely limited [12], [13], the remainder of the *T. brucei* complex exhibits relatively high variation, most of which does not partition into neatly defined geographic or taxonomic units [14], [15], [16], [17]. Thus, characterizing the genetic differences that contribute to a critical epidemiological trait such as human infectivity requires that those differences be evaluated in a comprehensive geographical and taxonomic context.

In the present study, we tested if the five non-synonymous substitutions previously hypothesized to alter HpHbR activity in *Tbg*1 [11] are both conserved in *Tbg*1 isolates and also absent from other *T. brucei* subspecies by examining HpHbR gene variation in *T. brucei* s.l. sampled across the entire range of the species complex. By narrowing the pool of substitutions that are specific to *Tbg*1, we expect to facilitate future functional studies aimed at understanding the contribution of HpHbR to conferring human serum resistance.

## Methods

### Sampling

Isolates of *Tbb*, *Tbg*1, *Tbg*2 and *Tbr*, were selected to incorporate representative genetic diversity from the entire geographic range of the *T. brucei* complex (Table S1, Figure 1b). When available, we included isolates of all co-occurring taxa from each country sampled (Figure 1b).

### PCR amplification and sequencing

For each isolate, DNA was extracted as described in [17]. PCR was performed using primers designed from *Tbb* (TREU927) and *Tbg*1 (Dal972) TriTrypDB database sequences (Tb927.6.440 and Tbg972.6.120, respectively) to amplify a 1297 base pair (bp) fragment that encompassed the entire *HpHbR* gene (HpHbR_F 5′ CGGGAAAGTTGTACGCAAG, HpHbR_R2 5′ CGACCACTTAATGTTACGAGG). For each PCR, 2–4 µL of a 1∶10 dilution of DNA extract were used. PCR reactions were performed using the reagents provided with GoTaq® DNA Polymerase and Green Master Mix. Difficult templates were amplified using Failsafe PCR 2X PreMixes Buffers (Epicentre Biotechnologies, Madison WI). All PCR reactions used the following cycle: Initial denaturation 95°C for 2 min, 50 cycles of 95°C for 35 s, 58°C for 35 s, and 72°C for 1 min 20 s and a final extension at 72°C for 7 min. PCR success was verified with 1% agarose gel electrophoresis. PCR products were purified and then sequenced (Yale DNA Analysis Facility) using two internal primers located in the middle of the sequence (HpHbR_F2in 5′ TGCTCGAGATATTCCTCAAG, HpHbR_Rin 5′ CTCCCACTGAAGCATTAGAC). The sequenced fragment included 22 nucleotides upstream of the *HpHbR* start codon, the entire HpHbR gene and 62 nucleotides downstream of the *HpHbR* stop codon.

### Sequence analysis and phasing of alleles

Sequences generated using the internal primers overlapped by approximately 200 bp permitting the assembly of an entire contiguous sequence of the *HpHbR* gene. Contiguous sequences were constructed and chromatograms from each isolate were manually examined for double peaks using the CLCBio DNA Workbench 5.7 (Cambridge, MA). Sites with double peaks were assigned the appropriate nucleotide ambiguity code. Sequences were aligned manually using MacClade 4.08 [18].

Samples with double peaks were considered heterozygotes. We used the programs SeqPhase [19] to format files and PHASE 2.1.1 [20] to resolve individual alleles from heterozygous sequences. To assess evidence for recombinant alleles and to relax the assumption of a stepwise mutation model, we employed the recombination model (MR) and the parent-independent models, respectively. Each run used 1000 iterations and a burnin of 500 generations and thinning interval = 1. The dataset was run twice with different random starting seeds and checked for consistency. The replicate with the best average goodness-of-fit was selected for subsequent analyses.

### Phylogenetic analysis and amino acid alignment

Nucleotide DNA sequence alignments were generated from phased alleles in MacClade 4.08. Haplotype networks were constructed in the program TCS [21]. DNA sequences were translated to amino acids and aligned in MacClade 4.08. Non-coding regions were removed from sequences and amino acid sequences were compared to those generated by [11].

## Results

### Sampling and allelic phase inference

We collected 1296 bp of sequence from each of 65 *T. brucei* isolates: 32 from *Tbb*, 15 from *Tbg*1, five from *Tbg*2 and 13 from *Tbr*. In addition, we generated sequence for one isolate each of *Trypanosoma equiperdum* and *Trypanosoma evansi* (Table S1), both of which are also members of the subgenus *Trypanozoon* but are not human infective (reviewed in [3]).

Of the 67 isolates sequenced in this study, 30 were heterozygous at the locus sequenced. PHASE 2.1.1 inferred a total of 34 alleles present in the 67 isolates. For all heterozygotes, allele pairs had Bayesian posterior probabilities of 1.0 across replicate runs, indicating that no alternative allele sequences could be inferred from the heterozygotes.

### Nucleotide variation within and among *T. brucei* strains

The 34 alleles recovered in this study exhibited a total of 40 variable sites, of which four were located outside the *HpHbR* coding region. Each of these four sites occurred in a distinct allele (f2, c, u3, z1) across a total of five isolates (Boula (*Tbg*1), STIB338 (*Tbr*), STIB386 (*Tbg*2), STIB777AE (*Tbb*), and KP13 (*Tbb*)). The remaining 36 variable sites were found within the coding region of *HpHbR* (File S1).

Most allelic diversity (28 alleles) was found in isolates of *Tbb*, *Tbr* and *Tbg*2 and much of this diversity was common to two or more taxa. Five of the seven alleles recovered from *Tbr* were identical to those found in *Tbb*. Likewise, four of the five alleles recovered from *Tbg*2 were also identical to alleles in *Tbb*. The most common allele in this study (u1; Table S1) was recovered from *Tbb*, *Tbr* and *Tbg*2. In contrast to these observations, we recovered four distinct alleles from *Tbg*1, but none of these were shared with any member of the subgenus *Trypanozoon*. Allelic diversity in *Tbg*1 was relatively low. Allele z1 (identical to the *Tbg1* sequence reported in Kieft et al. [2010]) was the most common *Tbg1* variant and was recovered from 26 of 30 sampled chromosomes. The remaining *Tbg*1 alleles differed by only one nucleotide from this common variant, z1. *Trypanosoma equiperdum* sequences were more similar to *Tbb* and *Tbr* sequences, though both alleles from *T. equiperdum* were unique. In *T. evansi*, alleles were identical to the most common allele found in *Tbb*, *Tbr* and *Tbg*2 (Fig. 1b).

### Protein coding differences inferred from nucleotide sequence

The HpHbR protein consists of 403 amino acids. In silico translation of the DNA sequences of the 34 alleles described above yielded 25 unique protein sequences (Figure 1, Figure S1). Notably, the single nucleotide difference in *HpHbR* that distinguished all isolates of *Tbg*1 from all other *T. brucei* isolates sampled in this study was non-synonymous, resulting in the replacement of leucine with serine at position 210 (L210S; Figure 1b, Figure S1). With one exception, all *Tbg*1 isolates possessed two copies of *HpHbR* that coded for just this single amino acid sequence (Z). In the exception, isolate ITMAP020578, one allele coded for amino acid sequence Z and the second allele coded for a second peptide (Y) differing from Z by just one amino acid at position 212. All other variation in HpHbR amino acid sequences partitioned to differences within and among *Tbb*, *Tbr* and *Tbg*2.

## Discussion

The primary goal of this study was to examine the genetic diversity of *HpHbR* in a broad geographical and taxonomic context to better characterize the mutations that potentially give rise to differences in HpHbR function and that may contribute to the phenotype of human serum resistance observed in *Tbg*1. An earlier study of *HpHbR* genetic diversity in a limited sample of parasite isolates identified five non-synonymous substitutions shared by *Tbg*1, but not found in *Tbb*, suggesting that these five differences could play an important functional role [11]. By sampling more broadly across the subgenus *Trypanozoon* and across Africa, we have demonstrated that just one of these substitutions (L210S) is conserved in *Tbg*1 and also absent from the most closely related trypanosome taxa, all of which are either susceptible to human serum (*Tbb*) or known to possess an alternative resistance mechanism (*Tbr* or *Tbg*2). Although our sample size remains relatively limited compared to the vast number of parasites distributed widely across Africa, the extremely low genetic diversity observed in *Tbg*1 HpHbR is consistent with prior population genetic studies [12], [13], [17] and we hypothesize that the mutation L210S is likely fixed in the taxon. This could be extended to field-circulating *Tbg*1 by using either allele specific PCR primers or a restriction fragment length polymorphism (RFLP) that targets the single nucleotide substitution (e.g., enzyme *Ple*I).

To the extent that the unique substitution in *Tbg*1 *HpHbR* prevents the uptake of TLF-1, this single amino acid change may play a key role in conferring serum resistance to this parasite. A role for HpHbR in facilitating lytic activity of human serum was originally established by experiments demonstrating that loss of HpHbR in *Tbb* (through RNA interference or gene knockout) conferred resistance to TLF-mediated lysis [22]. Later work demonstrated that *Tbb* selected to be TLF-1-resistant exhibited reduced HpHbR expression. Furthermore, the ectopic expression of *Tbg1* HpHbR (using an allele identical to the most common *Tbg1* allele identified in our study) in these serum resistant *Tbb* was not sufficient to restore human serum susceptibility, providing evidence for the altered function of *Tbg*1 HpHbR [11]. Our data indicate that this altered function likely stems from the L210S mutation in *Tbg*1, a substitution that effects an approximate 20-fold reduction in the affinity of HpHbR for HpHb [23]. Given that L210S appears to be the single mutation that distinguishes *Tbg*1 *HpHbR* from the *HpHbR* of all closely related members of the *Trypanozoon* subgenus, we hypothesize that this single mutation could play a major role in the serum resistance of *Tbg*1. However, this mutation is unlikely to be the sole factor. As noted previously, reduced expression levels of *HpHbR* are also likely to play a role in *Tbg*1 serum resistance [10], [11]. Also, while HpHbR is likely to be the main route of entry into the cell for TLF-1, poorly characterized alternative routes appear to exist for both TLF-1 and TLF-2, a second HDL particle that also exhibits trypanolytic activity [6]. Finally, an *in vitro* study has demonstrated that, regardless of receptor function, *Tbg*1 may be inherently resistant to apoL-1, the active trypanolytic factor in human serum [10]. While HpHbR may only be one component of *Tbg*1 serum resistance, the possible benefit of designing new drugs targeted to this receptor variant warrants further functional study to fully circumscribe its effect on serum resistance.

In contrast to *Tbg*1, the mechanism of *Tbg*2 resistance to human serum is thought to be independent of HpHbR, based on the finding that HpHbR from *Tbg*2 internalizes TLF-1 at a rate similar to that observed in *Tbb* and *Tbr* [10]. While that study included just a single strain of *Tbg*2 (STIB386), our results, which include data for several additional strains, suggest that this conclusion is likely to hold more broadly in *Tbg*2. Sequencing of *HpHbR* indicated that several isolates of *Tbg*2 shared sequence identity with isolates of both *Tbb* and *Tbr*, while exhibiting no overlap with isolates of *Tbg*1, a result that is consistent with previous surveys of neutral genetic markers [13], [17]. The genetic similarity of *HpHbR* observed among a large collection of isolates of *Tbb*, *Tbr*, and *Tbg*2 suggests that the function of HpHbR in *Tbg*2 is more likely to reflect that of *Tbb* and *Tbr* than *Tbg*1 and further supports the conclusion that *Tbg*2 serum resistance is independent of HpHbR. Our study surveyed only five strains of *Tbg*2, but even these five strains exhibited substantially more diversity than *Tbg*1 at both the nucleotide and amino acid level. The genetic variability of *HpHbR* in *Tbg*2 reiterates the fact that *Tbg*2, unlike *Tbg*1, is not genetically homogeneous and suggests that future studies should consider this diversity when examining functional differences among parasite subgroups.

## Supporting Information

Figure S1 **Amino acid alignment of the complete HpHbR gene.** Amino acids are identified by a unique color and a single letter abbreviation at the top of the figure. Letters at left identify unique amino acid sequences shown in Figure 1b. An asterisk (*) indicates that multiple isolates share the same amino acid sequence (see Table S1). Variable positions are highlighted below the alignment.
(EPS)

File S1 **This file contains the DNA sequences from each of the alleles identified in this study.** Allele sequences were inferred from direct sequences *in silico* using the program PHASE 2.1.1 (Stephens et al. 2001).
(FAS)

Table S1 **Subgenus** ***Trypanozoon*** **isolate taxonomic classification, collection information and characterization of genetic variation at the** ***HpHbR*** **locus.**
(DOCX)
