# Supplementary material for: Trypanosoma brucei gambiense Group 1 Is Distinguished by a Unique Amino Acid Substitution in the HpHb Receptor Implicated in Human Serum Resistance
Source: PLoS Negl Trop Dis. 2012 Jul 10;6(7):e1728. doi: 10.1371/journal.pntd.0001728 (PMC3393672; doi:10.1371/journal.pntd.0001728)
Supplement: Table S1 — Subgenus Trypanozoon isolate taxonomic classification, collection information and characterization of genetic variation at the HpHbR locus. (DOCX) [file pntd.0001728.s003.docx]

Tables

Table S1. Subgenus *Trypanozoon* isolate taxonomic classification, collection information and characterization of genetic variation at the *HpHbR* locus.

| **Subspecies^a^** | **Isolate code^b^** | **Synonymous name(s)** | **Isolate origin** | **Host^c^** | **Year** | **DNA alleles^c^** | **Inferred a.a. sequence^c^** | **GenBank Accession Number^d^** |
| --- | --- | --- | --- | --- | --- | --- | --- | --- |
| *Tbb***^e^** | ob153 | P16F | Cameroon, Fontem | pig | 1999 | u1/u1 | U/U | JX143824 |
| *Tbb***^e^** | ob154 | P7F | Cameroon, Fontem | pig | 1999 | u1/u1 | U/U | JX143832 |
| *Tbb***^e^** | ob155 | P8F | Cameroon, Fontem | pig | 1999 | u1/u1 | U/U | JX143833 |
| *Tbb* | SW3/87 | SW3/87 | D. R. Congo, Bandundu | pig | 1987 | r/d | R/D | JX143831 |
| *Tbb***^e^** | ob152 | TSW 65 (KP1) | Ivory Coast, Vavoua | pig | 1982 | t2/w | T/W | JX143823 |
| *Tbb* | TSW 187/78E | TSW 187/78E | Ivory Coast | pig | 1978 | s/u2 | S/U | JX143829 |
| *Tbb* | TSW 196 | TSW 196 | Ivory Coast | pig | 1978 | u1/u1 | U/U | JX143830 |
| *Tbb* | PTAG 129 | PTAG 129 | Ivory Coast, Daloa | pig | 1985 | t1/u1 | T/U | JX143826 |
| *Tbb* | PTAG 130 | PTAG 130 | Ivory Coast, Daloa | pig | 1985 | u1/u1 | U/U | JX143827 |
| *Tbb* | KP14 clone 1 | KP14 clone 1 | Ivory Coast, Kouassi-Perita | *Glossina palpalis palpalis* | 1989 | u1/u1 | U/U | JX143812 |
| *Tbb* | KP14 clone 2 | KP14 clone 2 | Ivory Coast, Kouassi-Perita | *G. p. palpalis* | 1989 | u1/u1 | U/U | JX143809 |
| *Tbb* | KP33 clone 8 | KP33 clone 8 | Ivory Coast, Kouassi-Perita | *G. p. palpalis* | 1989 | u1/u1 | U/U | JX143810 |
| *Tbb* | KP13 clone2 | KP13 clone2 | Ivory Coast, Kouassi-Perita | *G. p. palpalis* | 1989 | u3/u3 | U/U | JX143811 |
| *Tbb* | cp003 | TREU 927/4 | Kenya, Kiboko | *G. pallidipes* | 1970 | m/m | M/M | JX143803 |
| *Tbb* | cp019 | M249 | Kenya, Matuga | sheep | 1981 | m/q | M/Q | JX143804 |
| *Tbb* | B8/18 clone B | B8/18 clone B | Nigeria, Nsukka | pig | 1962 | u1/u1 | U/U | JX143802 |
| *Tbb* | cp022 | STIB 215 | Tanzania, Serengeti NP | lion | 1971 | m/p1 | M/P | JX143805 |
| *Tbb* | ob010 | STIB 920; STIB 246; STIB 348 | Tanzania, Serengeti NP | kongoni | 1971 | f1/b1 | F/B | JX143814 |
| *Tbb* | ob059 | STIB 205 | Tanzania, Serengeti NP | lion | 1971 | p1/p1 | P/P | JX143817v |
| *Tbb* | ob068 | STIB 204 | Tanzania, Serengeti NP | lion | 1971 | k/i | K/I | JX143818 |
| *Tbb* | ob071 | STIB 210 | Tanzania, Serengeti NP | lion | 1971 | p1/p1 | P/P | JX143819 |
| *Tbb* | ob087 | STIB 247 | Tanzania, Serengeti NP | kongoni | 1971 | f1/b1 | F/B | JX143820 |
| *Tbb* | ob089 | S42-030; STIB 366 Clone1 | Tanzania | warthog | 1966 | b1/b1 | B/B | JX143821 |
| *Tbb* | ob178 | RUMP501 | Tanzania | cf. cattle | 1956 | u1/h | U/H | JX143825 |
| *Tbb* | ob009 | STIB 777AE | Uganda, Busoga | *G. fuscipes* | 1971 | n/c | N/C | JX143813 |
| *Tbb* | ob030 | KETRI 1956; STIB 776 | Uganda, Busoga | *G. fuscipes* | 1971 | h/f1 | H/F | JX143815 |
| *Tbb* | ob051 | STIB 340 | Uganda | hippo | 1961 | f1/f1 | F/F | JX143816 |
| *Tbb* | ob091 | EATRO 1244; LUMP1026; STIB 783 | Uganda | *G. pallidipes* | 1969 | f1/b1 | F/B | JX143822 |
| *Tbb* | tRE087 |  | Uganda, Arua | cow | 2001 | v/x | V/X | JX143828 |
| *Tbb* | cp024 | TRPZ 239 | Zambia, Luangwa valley | giraffe | 1982 | e/e | E/E | JX143806 |
| *Tbb* | cp026 | TRPZ 286 (pop 1) | Zambia, Luangwa valley | *G. pallidipes* | 1983 | l/j | L/J | JX143807 |
| *Tbb* | cp027 | TRPZ 317 (clone 4) | Zambia, Luangwa valley | *G. morsitans* | 1983 | g/f1 | G/F | JX143808 |
| *Tbg*1 | ob048 | 001 K1 Angola | Angola | human | 1998 | z1/z1 | Z/Z | JX143840 |
| *Tbg*1 | Boula | Boula | Congo, Bouenza | human | 1989 | z3/z3 | Z/Z | JX143837 |
| *Tbg*1 | Tad | Tad | Congo, Bouenza | human | 1989 | z1/z1 | Z/Z | JX143848 |
| *Tbg*1 | ob126 | SOMABc | Cameroon, Campo | human | 1999 | z1/z1 | Z/Z | JX143845 |
| *Tbg*1 | A004 | A004 | Cameroon, Fontem | human | 1988 | z1/z1 | Z/Z | JX143836 |
| *Tbg*1 | Fontem strain 10 | Fontem strain 10 | Cameroon, Fontem | human | 1989 | z1/z1 | Z/Z | JX143838 |
| *Tbg*1 | 1898 | ITMAP1898 | D. R. Congo | human | 1974 | z1/z2 | Z/Z | JX143835 |
| *Tbg*1 | ob186 | ITMAP020578/Ntuma | D. R. Congo, Bandundu/Lebu | human | 1977 | z1/y | Z/Y | JX143846 |
| *Tbg*1 | ob202 | ITMAP210879/Moer82 | D. R. Congo, Bas Congo/Kwilu-Ngongo | human | 1970 | z1/z1 | Z/Z | JX143847 |
| *Tbg*1 | 1829 (Aljo) | ITMAP1829 | D. R. Congo, Bandundu | human | 1970 | z1/z1 | Z/Z | JX143834 |
| *Tbg*1 | ob080 | DAL 1086 R | Ivory Coast, Daloa | human | 1987 | z1/z1 | Z/Z | JX143841 |
| *Tbg*1 | ob083 | TH Dal 069; STIB 717 | Ivory Coast, Daloa | human | 1978 | z1/z1 | Z/Z | JX143842 |
| *Tbg*1 | ob007 | STIB930; TH1/78E (031) | Ivory Coast, Vavoua | human | 1978 | z1/z1 | Z/Z | JX143839 |
| *Tbg*1 | ob107 | K00014JD | Sudan, Western Equatoria, Lazoh, Midi | human | 2003 | z1/z1 | Z/Z | JX143843 |
| *Tbg*1 | ob111 | K0303045 | Sudan, Western Equatoria, Nyau 1 | human | 2003 | z1/z1 | Z/Z | JX143844 |
| *Tbg*2 | ob032 | TH 114/78E(020); STIB 386 | Ivory Coast, Koudougou | human | 1978 | n/c | N/C | JX143813 |
| *Tbg*2 | ob146 | TH 113/78E(020) | Ivory Coast, Koudougou | human | 1978 | t1/t3 | T/T | JX143851 |
| *Tbg*2 | ob151 | TH 2/78E(020) | Ivory Coast, Koudougou | human | 1978 | u1/u1 | U/U | JX143852 |
| *Tbg*2 | HTAG 15/5 | IPR 15/5 | Ivory Coast, Tagoura | human | 1985 | u1/u1 | U/U | JX143849 |
| *Tbg*2 | TH126 | TH126 (020) | Ivory Coast, Koudougou | human | 1978 | u1/u1 | U/U | JX143853 |
| *Tbr* | ob018 | STIB 338; Dunnels strain | Botswana, Mababe, Ngamiland | human | 1960 | f2/f2 | F/F | JX143854 |
| *Tbr* | ob095 | EATRO 1192; STIB 809; Gambela 1 | Ethiopia, Illubabor | human | 1967 | u1/u1 | U/U | JX143863 |
| *Tbr* | ob078 | EATRO 0237 | Kenya, Bungala | human | 1961 | h/f1 | H/F | JX143861 |
| *Tbr* | ob012 | EATRO 1836; STIB 056 | Tanzania, Serengeti NP | waterbuck | 1971 | u1/u1 | U/U | JX143855 |
| *Tbr* | ob054 | STIB 316; STIB 236 | Tanzania, Serengeti NP | lion | 1971 | f1/b1 | F/B | JX143857 |
| *Tbr* | ob056 | STIB 286; STIB 243 | Tanzania, Serengeti NP | hyaena | 1971 | f1/b1 | F/B | JX143858 |
| *Tbr* | ob065 | EATRO 1873; STIB 262 | Tanzania, Serengeti NP | kongoni | 1970 | i/b1 | I/B | JX143859 |
| *Tbr* | ob066 | EATRO 1873; STIB 263 | Tanzania, Serengeti NP | kongoni | 1970 | i/b1 | I/B | JX143860 |
| *Tbr* | ob093 | STIB 364-A; STIB241 | Tanzania, Serengeti NP | lion | 1971 | f1/b1 | F/B | JX143862 |
| *Tbr* | ob021 | ETat 10; ILRAD 853; TREU 164; STIB 391 | Uganda, Lugala | *G. pallidipes* | 1960 | u1/f1 | U/F | JX143856 |
| *Tbr* | ob096 | UTRO 030790; STIB 848 | Uganda | human | 1990 | u1/f1 | U/F | JX143864 |
| *Tbr* | ob098 | UTRO 120890A; STIB 851 | Uganda | human | 1990 | u1/f1 | U/F | JX143865 |
| *Tbr* | ob156 | TRPZ 166 | Zambia, Kakumbi | ox | 1982 | f1/f1 | F/F | JX143866 |
| *Teq* | ob105 | ITMAS 241199C; OVI | South Africa |  |  | o/p2 | O/P | JX143867 |
| *Tev* | ob106 | ITMAS 060100; Rotat1.2 | Indonesia | water buffalo | 1982 | u1/u1 | U/U | JX143868 |
| *Tbb** | Lister 427* | Lister 427 | Uganda | sheep | 1960 | not available | A/A |  |
| *Tbb** | 927/4* | TREU 927 |  |  |  | m/m | M/M |  |
| *Tbg*1*** | 972* | Dal 972 | Ivory Coast, Daloa | human | 1986 | z1/z1 | Z/Z |  |
| *Tbg*1**** | ELIANE** | Eliane | Ivory Coast | human | 1952 | z1/z1 | Z/Z |  |

^a^, Sub-species assignment based on disease-symptoms and phylogeographic analysis from Balmer et al. (2011). *Tbb*, *T. b. brucei*; *Tbg1*, *T. b. gambiense* group 1; *Tbg2*, *T. b. gambiense* group 2; *Tbr*, *T. b. rhodesiense; Teq, T. equiperdum; Tev, T. evansi.* An asterisk (*) indicates data from Kieft et al. (2010) and two asterisks (**) indicate data from Capewell et al. (2011). DNA sequence was not available for *Tbb* LISTER 427 from Kieft et al. (2010).

^b^, Isolate codes correspond to those in Balmer et al. (2011). Names starting with “ob” correspond to isolates “b” and those starting with “cp” to “c” in Balmer et al. (2011).

^c^, The two DNA alleles recovered from each isolate are identified with lower case letters corresponding to inferred amino acid (a.a.) sequences described in Figure 1b (e.g., an isolate typed as u1/u1 is homozygous for one of three alleles that codes for amino acid U).

^d^, GenBank Accesion numbers will be provided upon acceptance of the manuscript.

^e^ Listed as *T. brucei* non-gambiense group 1“ by Nkinin et al. (2002).
